# Simultaneous circulation of two West Nile virus lineage 2 clades and Bagaza virus in the Zambezi region, Namibia

**DOI:** 10.1371/journal.pntd.0009311

**Published:** 2021-04-02

**Authors:** Heiko D. Guggemos, Matthias Fendt, Christian Hieke, Verena Heyde, John K. E. Mfune, Christian Borgemeister, Sandra Junglen

**Affiliations:** 1 Institute of Virology, Charité - Universitätsmedizin Berlin, corporate member of Free University Berlin, Humboldt-University Berlin, and Berlin Institute of Health, Germany; 2 German Center for Infection Research (DZIF), Berlin, Germany; 3 Department of Biological Sciences, University of Namibia, Windhoek, Namibia; 4 Center for Development Research (ZEF), University of Bonn, Bonn, Germany; Connecticut Agricultural Experiment Station, UNITED STATES

## Abstract

Flaviviruses include a great diversity of mosquito-borne arboviruses with epidemic potential and high global disease burden. Several flaviviruses are circulating in southern Africa affecting humans and livestock, among them West Nile virus (WNV) and Wesselsbron virus. Despite their high relevance, no arbovirus surveillance study has been conducted for more than 35 years in Namibia. In this study we assessed the diversity of flaviviruses circulating in mosquitoes in the densely populated, semi-tropical Zambezi region of north-eastern Namibia. In total, 10,206 mosquitoes were sampled in Bwabwata and Mudumu national parks and Mashi and Wuparo conservancies and screened for flavivirus infections. A high infection rate with insect-specific flaviviruses was found with 241 strains of two previously known and seven putative novel insect-specific flaviviruses. In addition, we identified ten strains of WNV in the main vector *Cx*. *univittatus* sampled in the Mashi conservancy. Surprisingly, the strains fell into two different clades of lineage 2, 2b and 2d. Further, three strains of Bagaza Virus (BAGV) were found in *Cx*. *univittatus* mosquitoes originating from Mudumu national park. Assessment of BAGV growth in different cell lines showed high replication rates in mosquito and duck cells and about 100,000fold lower replication in human, primate and rodent cells. We demonstrate a wide genetic diversity of flaviviruses is circulating in mosquitoes in the Zambezi region. Importantly, WNV and BAGV can cause outbreaks including severe disease and mortality in humans and birds, respectively. Future studies should focus on WNV and BAGV geographic distribution, as well as on their potential health impacts in and the associated social and economic implications for southern Africa.

## Author summary

Mosquitoes serve as vectors for the transmission of infectious diseases. Some of the most important mosquito-borne arboviruses belong to the genus *Flavivirus*, which can induce severe disease in humans and livestock. Surveillance of vector populations provide information on circulating arboviruses and may help to identify local outbreaks. Here we sampled mosquitoes over three wet seasons in the densely populated, semi-tropical Zambezi region of north-eastern Namibia and tested them for infections with flaviviruses. We observed simultaneous circulation of two different West Nile virus clades in the main vector species *Cx*. *univittatus*. Humans infected with West Nile virus can develop flu-like symptoms or in rare cases meningoencephalitis. Further, we detected Bagaza virus in *Cx*. *univittatus* from another locality and season. Bagaza virus infects birds leading to high mortality rates and may also infect humans. Our data suggest that both viruses are endemic in the Zambezi region and may affect human health and well-being in Namibia.

## Introduction

The genus *Flavivirus* within the family *Flaviviridae* contains some of the most dangerous and life-threatening mosquito-borne viruses like Dengue virus, Yellow fever virus, Japanese encephalitis virus, Zika virus and West Nile virus (WNV) [1]. Land use and socio-economic changes, as well as global trade and travel fueled the spread of these and other flaviviruses to new geographic regions where they caused epidemics and often became endemic [2]. For instance, Dengue virus is now endemic in >100 countries and affects about 400 million people each year [3].

Several flaviviruses are endemic in southern Africa, with WNV and Wesselsbron virus (WSLV) among the most prevalent ones [4]. WNV is a mosquito-borne flavivirus that was first isolated in 1937 from a patient with febrile symptoms in the West Nile district of Uganda [5]. The virus is maintained in nature in a mosquito-bird transmission cycle where it can be transmitted to humans and horses by a bite of an infected mosquito. WNV is geographically the most widely distributed encephalitic flavivirus and has caused outbreaks in the Americas, Africa, Europe and Asia [6–10]. Approximately 80% of human infections with WNV are asymptomatic. Symptomatic patients usually show flu-like symptoms, such as fever, fatigue, headache, nausea, muscle pain and rash [11,12]. Less than 1% of WNV patients develop severe symptoms, such as meningoencephalitis that can lead to death [13]. WNV can cause sporadic outbreaks, usually after heavy rainfall, resulting in a sharp vector population build-up [14]. In 1974, the Karoo and Northern Cape provinces in South Africa experienced a large WNV outbreak that affected ten thousand of humans though no cases of encephalitis and no deaths were recorded [15–17]. In South Africa WNV infections have been observed in a range of wild and domesticated animals and seroprevalence rates of 24%, 18% and 30% against WNV were detected in humans, cattle, and sheep, respectively using an *in-house* ELISA indicating that flaviviruses are highly prevalent across the country [18,19].

For WSLV, first isolated in the South African town of Wesselsbron in 1955, similar seroprevalence rates in humans and cattle were observed in South Africa [18,20]. WSLV causes arthralgia, myalgia and fever in humans [21,22]. Symptoms in cattle and sheep are more severe and similar to Rift Valley fever with abortion rates of up to 20% [23–25].

Bagaza virus (BAGV) is a mosquito-borne flavivirus pathogenic to birds and has only recently been detected in dead monal pheasants near Pretoria in South Africa [26]. Bagaza virus shares high sequence similarity with Israel turkey meningoencephalitis virus (ITV), which was first isolated in 1958 from domesticated turkeys in Israel [27] and was detected in South African turkeys in 1978 [28]. Based on the high sequence identity, both viruses have been suggested to belong to the same species [29]. Typical symptoms in susceptible bird species include weight loss, weakness, and apathy with mortality rates as high as 30% in partridges and pheasants [30,31]. BAGV was first isolated in 1966 from *Culex* mosquitoes collected in Bagaza, Central African Republic [32]. Subsequently, it has been detected in mosquitoes collected in Senegal and Mauritania [33–35] and India [36]. BAGV neutralizing antibodies were later detected in encephalitic patients from India suggesting that the virus can also infect humans [36]. BAGV was first isolated from vertebrates in 2010 from dead red-legged partridges and pheasants in southern Spain [31].

Usutu virus (USUV) and Banzi virus (BANV) are also endemic in the region and were both first isolated close to the Usutu river in South Africa [37–39]. Finally, there are historic reports of DENV epidemics in southern Africa in the late 19^th^ and early 20^th^ century, with most recent reports from northern Mozambique dating back to 1984 [40]. Since then, only few reports on DENV in the region have been published, primarily originating from imported cases, and hence DENV is most likely not circulating in this part of the continent [41].

Little is known on the current incidence of flaviviruses in Namibia. WNV, WSLV, BANV, Spondwenni and Yellow fever have been detected in the local population in serosurveys across the north-eastern Zambezi region in the 1950s and 1960s with antibody prevalence rates of 12%, 23%, 9%, 4 and 2% in neutralization assays [42]. In the 1980s, rates of 29% WNV and 5% WSLV seroprevalence were recorded by hemagglutination inhibition assays in the same region [43], and WNV was detected in a febrile patient in Namibia’s Ovamboland in 1989 [44]. Since independence in 1990, only one mosquito-borne arbovirus surveillance study was conducted in Namibia, focusing on the hot and semi-arid central region of the country, with a seroprevalence rate not exceeding 8% against flaviviruses reported in human sera by ELISA testing [45]. Yet since the 1980s, no comparable data has been gathered for the rather densely populated, semi-tropical regions in the north of the country [45].

In this study we sought to assess the diversity of flaviviruses circulating in mosquitoes in the Zambezi region in north-eastern Namibia. To obtain an overview on the genetic diversity of endemic viruses, we sought to sample a broad diversity of mosquito species from various land-use types.

## Material and methods

### Ethics statement

A research permit to conduct this research was received from the National Commission on Research, Science and Technology (permit number RPIV00442018).

### Mosquito sampling and identification

**Fig 1:**
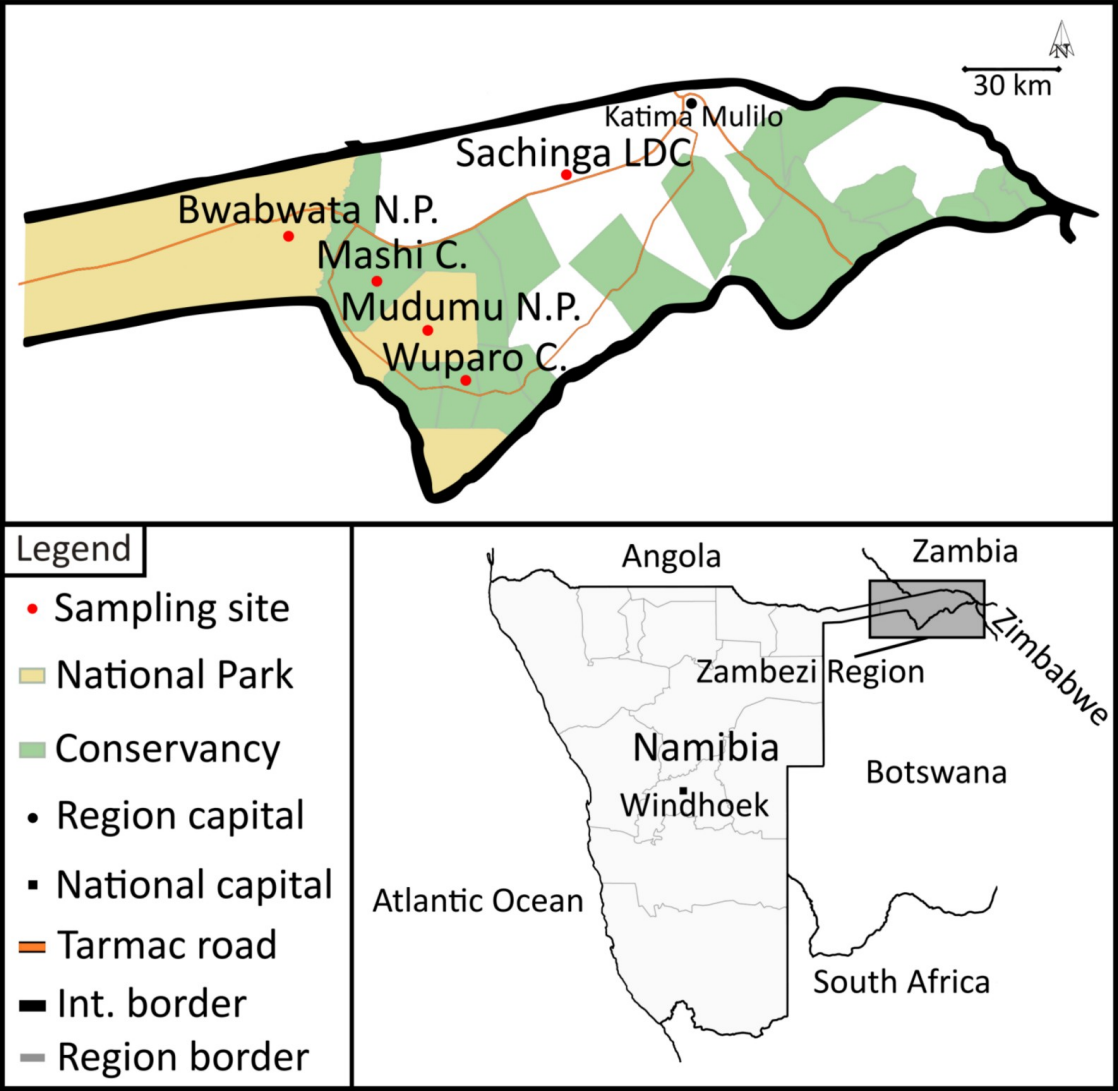
Schematic map of mosquito-sampling sites in Namibia. Samples were retrieved from Bwabwata and Mudumu National Parks, Wuparo and Mashi Conservancies and Sachinga Livestock Development Centre (Sachinga LDC). The map base layer is based on https://www.naturalearthdata.com, and the map’s details are based on [94].

Mosquitoes were sampled in north-eastern Namibia in the Sachinga Livestock Development Centre in May and June 2018, in Mudumu National Park and Wuparo Conservancy in November and December 2018, in Bwabwata National Park in February and March 2019, and in Mashi Conservancy in March and April 2019 and in February and March 2020 (Fig 1). Four sites each in the National Parks were selected to represent sites with medium and high pressure of elephants. In the conservancies, we selected four sites each in rangeland, agricultural field and in the conservancy core area to cover all existing land-use types of the conservancies. In total, two BG Sentinel traps (Biogents AG, Regensburg, Germany), two CDC Light traps and one CDC Gravid trap (both John W. Hock Company, Gainesville, United States of America) were operated from dusk till dawn for five consecutive days at each site (total trapping effort 1,480 trap nights). BG Sentinel traps were baited with BG-Lure (Biogents AG, Regensburg, Germany), 1-Octen-3-ol and worn socks on different days. The combination of trap types and attractants was used in order to sample a high diversity of mosquito species. Mosquitoes were stored in liquid nitrogen in the field. Mosquito species were identified morphologically in the laboratory using standard keys [46–48].

### Viral RNA extraction and PCR-screening

Individual mosquito specimens were homogenized in 500 μl phosphate-buffered saline (PBS) using ceramic beads and a Tissue Lyser (QIAGEN, Hilden, Germany) and subsequently arranged into pools of 10 according to species and sampling location using 100 μl of the individual mosquito homogenate. The rest of the mosquito homogenate was retained for further analysis based on individual specimens. RNA was extracted from 200 μl of pooled mosquito homogenates using the MagNA Pure 96 DNA and Viral NA Small Volume Kit (Roche Diagnostics, Mannheim, Germany). The RNA was eluted in 100 μl of provided elution buffer. Subsequently cDNA synthesis was performed using SuperScript IV reverse transcriptase (Thermo Fisher Scientific GmbH, Dreieich, Germany) and random hexamer primers (Integrated DNA Technologies Germany GmbH, Munich, Germany). Mosquito pools were screened for flaviviruses using generic PCR assays targeting the RNA-dependent RNA polymerase gene according to published protocols [49,50]. Nucleotide sequences were obtained by Sanger sequencing (Microsynth Seqlab GmbH, Göttingen, Germany), analysed in Geneious 9.1.8 (Biomatters ltd., Auckland, New Zealand) and compared to GenBank using the Basic Local Alignment Search Tool (BLAST) (https://blast.ncbi.nlm.nih.gov/Blast.cgi). In addition, all mosquito pools were tested for WNV [51], Dengue virus [52], Zika virus [53], Yellow fever virus [54] and BAGV (forward 5’-TCCAGGGAAGACCAGAGAGG-3’, reverse 5’-AGGCTTCAGCAATCCTTCCC-3’, probe 5’-FAM-TGCGTTGAACACCTTTACCA-ZEN-3’) using virus specific real-time-PCRs. RNA was extracted from single mosquito specimen of virus-positive pools and tested individually for infection using virus-specific RT-qPCRs as described above.

### Genome sequencing and analysis

A fragment of the viral E protein (1,336 nucleotides) of all detected WNV strains was amplified by nested PCR using the primers WNV-E F1 5’-GAGGGAGTGTCTGGAGCTAC-3’, WNV-E R1 5’-CTGTCACGGGCATTGATTCC-3’; WNV-E F2 5’-TGGGTTGATCTGGTACTGGA-3’, WNV-E R2 5’-GAAGTCCCTGTGTGATCCAG-3’. Full WNV genomes of two selected strains (M6848NA-2020 and M6646-NA-2020) were sequenced by amplifying overlapping PCR amplicons of 800–1,000 nucleotides in length using generic nested primers based on an alignment of WNV lineage 2 sequences (primer sequences will be published elsewhere). For full genome sequencing of the BAGV strain MP314-NA-2018 nested primer pairs were designed based on an alignment of all currently available BAGV genomes aiming to amplify overlapping fragments of approximately 1,500 nucleotides (S1 Table). Amplicons were sequenced by Sanger sequencing and genomes were assembled using closely related strains as reference genomes in Geneious 9.1.8 (Biomatters ltd., Auckland, New Zealand).

### Phylogenetic analyses

Nucleotide and amino acid sequences of RdRp and E genes, as well as entire open reading frames (ORFs) were aligned with related viral sequences in Geneious using the CLUSTAL W [55] and MAFFT algorithms [56]. Phylogenetic trees were inferred by the maximum-likelihood (ML) method with the best suitable substitution matrix as identified by MEGA-X [57] using PhyML [58]. The phylogeny of all detected flaviviruses was inferred using the GTR model with a fixed Gamma shape parameter of 0.36 and an estimated fraction of invariable sites. The phylogenetic relationship based on WNV E genes was inferred with the GTR model using a fraction of 0.46 invariable sites and a fixed Gamma shape parameter of 1.2. The phylogenetic relationship based on WNV full genomes was inferred with the JC69 model using an estimated fraction of invariable sites and an estimated Gamma shape parameter. The BAGV phylogeny based on full BAGV genomes was inferred using the TN93 model, a fixed Gamma shape parameter of 0.27, and an estimated fraction of invariable sites and the BAGV tree based on RdRP sequence fragments was inferred with the TN93 model, a fixed Gamma shape parameter of 0.34, and an estimated fraction of invariable sites. Confidence testing was performed based on 1,000 bootstrap iterations.

### Genetic barcoding for mosquito species identification and blood-meal analysis

From selected mosquito specimens a fragment of the cytochrome c oxidase I (COI) gene was amplified based on generic primers for invertebrates [59]. To identify the vertebrate feeding source of selected mosquitoes, a fragment of the COI gene was amplified using generic vertebrate specific primers [60]. Obtained PCR-products were sequenced by Sanger sequencing (Microsynth Seqlab GmbH, Göttingen, Germany) and compared to GenBank using BLAST (https://blast.ncbi.nlm.nih.gov/).

### Virus isolation in cell culture

For virus isolation attempts, mosquito (C6/36, *Aedes albopictus*; CXT, *Culex tarsalis)* and mammalian (VeroE6, African green monkey) cell lines were either inoculated with an aliquot of the virus-positive mosquito-pool or an aliquot of the individual virus-positive mosquito homogenate as described previously [61]. Cultures were checked daily for occurrence of a cytopathic effect (CPE). An aliquot of the supernatant (100 μl) was used for infection of fresh cells eight days post infection (passage 1). This procedure was repeated four times. The cell culture supernatants of each passage were in addition checked for viral replication by real-time PCR as described below.

Virus stocks were prepared from infectious cell culture supernatant of MP312-NA-2018 passage 4, MP314-NA-2018 passage 4, and MP370-NA-2018 passage 5. The number of infectious particles was determined by Tissue culture Infectious Dose 50 (TCID50) end-point dilution assay [62,63].

### Virus growth kinetics

Different vertebrate (VeroE6, African green monkey; BHK-21, hamster; KN-R, cattle; DF-1, chicken [64]; LMH, chicken [65]; AGE1.CR, duck [66]; HEK293, human) and insect (C6/36, *Aedes albopictus*; CXT, *Culex tarsalis)* cell lines were infected in duplicates at a multiplicity of infection (MOI) of 0.1 and 0.01, respectively. Aliquots of cell culture supernatants were sampled every 24 hours for five days and virus copy numbers were determined by virus specific RT-qPCR using a plasmid-based standard dilution series. Briefly, the 114 nucleotide BAGV qPCR amplicon was amplified by conventional PCR using infectious supernatant of MP314-NA-2018 and subsequently cloned into the TOPO TA vector using the TOPO TA cloning kit according to the manufacturer’s protocol (Thermo Fisher Scientific GmbH, Dreieich, Germany). An overnight culture of a positive clone was prepared in LB medium supplemented with ampicillin (100 μg/mL) and plasmid DNA was extracted with the QIAprep Spin Miniprep kit (QIAGEN, Hilden, Germany). Plasmid concentration was measured using a NanoDrop spectrophotometer (Thermo Fisher Scientific GmbH, Dreieich, Germany) and a tenfold serial dilution series from 1x10^1^ – 1x10^6^ genome copies/ml was used for quantification.

## Results

### Mosquito collection and flavivirus screening

**Fig 2:**
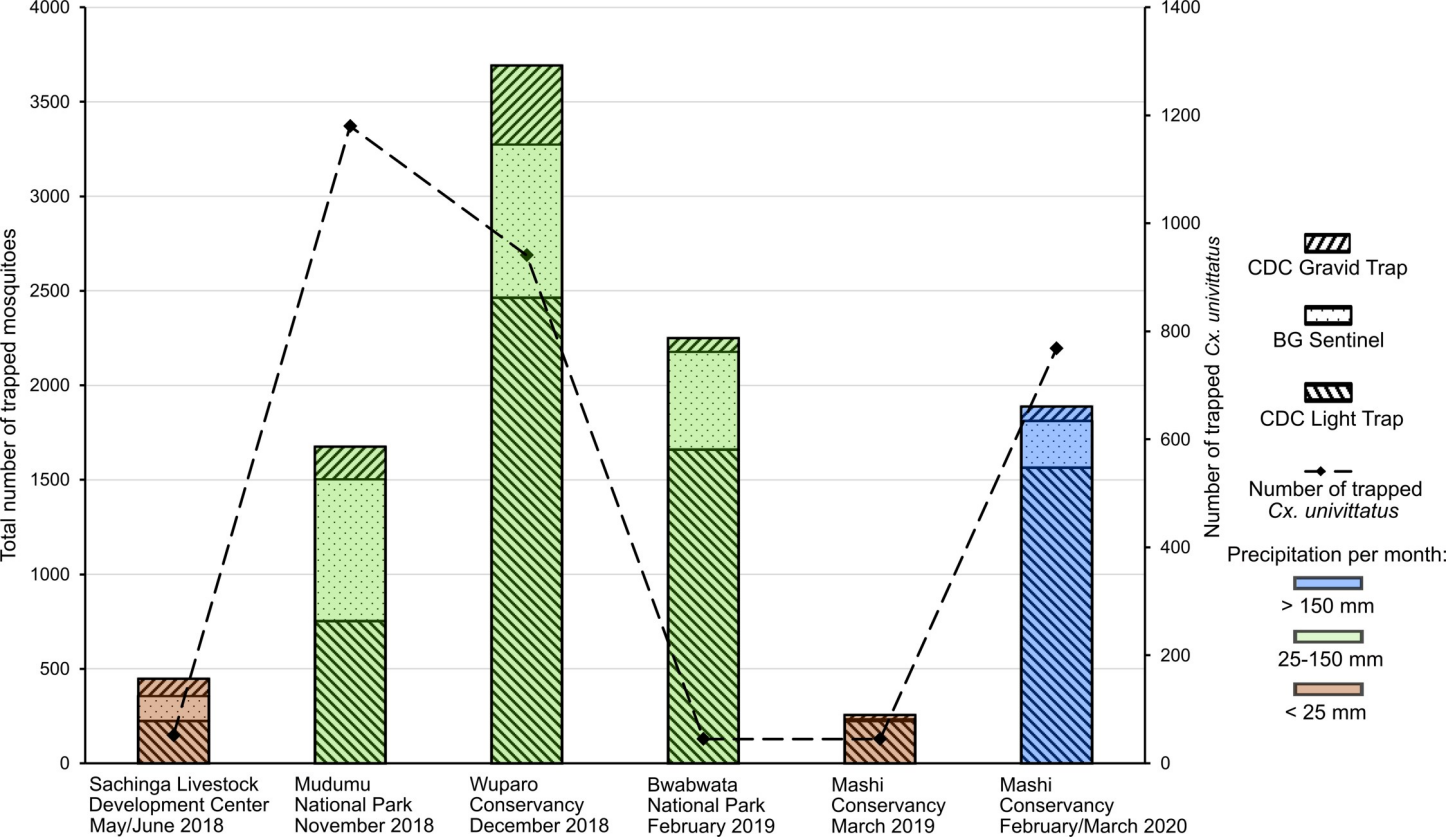
Overview of collected mosquitoes in the Zambezi region, Namibia. Number of sampled mosquitoes per locality and collection time is given by bar charts according to trap type and amount of rainfall at the time of collection [69]. Number of collected *Cx*. *univittatus* mosquitoes per locality is shown by a dashed line.

A total of 10,206 mosquitoes were collected in national parks and conservancies in the Zambezi region, Namibia, from May 2018 to March 2020 (**Fig 1**). In total, 42 different species were collected with *Culex univittatus* (3032, 30%), *Coquillettidia metallica* (1851, 18%) and *Mansonia uniformis* (1652, 16%) as the most abundant species. About three quarters of the sample set represented female (n = 7840, 76.8%) and about one quarter male mosquitoes (n = 2181, 21.4%) and for 185 (1.8%) mosquitoes the sex could not be identified due to poor sample condition. The average catch size was 4.3 mosquitoes per catch bag. Most of the mosquitoes were collected in the Wuparo conservancy and low catch rates were associated with low precipitation rates (Fig 2). After individual homogenization, all mosquitoes were sorted into 1,021 pools, comprising 10 individuals each.

Screening of all pools with a generic flavivirus RT-PCR yielded 246 viral RdRP sequences falling in eleven clusters. Two sequences from two pools of female *Cx*. *univittatus* mosquitoes showed 96.9% and 97.9% identity to the South African WNV strains HM147822 and EF429197, respectively, and three sequences from *Culex* sp. mosquitoes showed 99.3–100% identity to BAGV indicating the detection of WNV and BAGV in mosquitoes from Namibia. The term strain is in the following referred to as a variant of a given virus species. All other 241 sequences showed identities to insect-specific flaviviruses (ISF). We detected 10 strains of Culex flavivirus in *Culex* spp. mosquitoes and 60 strains of Mosquito flavivirus AMH010516 in *Mansonia* spp. and *Cx*. *univittatus* mosquitoes (Table 1). The other 171 sequences assembled in seven groups which showed pairwise identities of 70–77% to known ISF suggesting that they most likely belong to seven distinct species (Table 1). In addition, all mosquito pools were tested negative for DENV, ZIKV and YFV in specific qPCR assays.

**Table 1:** Detected insect-specific flaviviruses in the Zambezi region, Namibia.

Virus	Reference sequence	Mosquito host (number of detections)
Culex flavivirus	MP572-NA-2018	*Culex sp*. *(7)*, *Cx*. *nebulosus (1)*, *Cx*. *univittatus (1)*, *Cx*. *quinquefasciatus (1)*
Mosquito flavivirus AMH010516	MP76-NA-2018	*Mansonia*. *uniformis (58)*, *Ma*. *africana (1)*, *Cx*. *univittatus (1)*
Unassigned	MP74-NA-2018	*Coquillettidia metallica (86)*, *Cx*. *univittatus (1)*
Unassigned	MP7-NA-2018	*Cq*. *metallica (10)*
Unassigned	MP484-NA-2018	*Ma*. *uniformis (38)*, *Ma*. *africana (2)*, *Cq*. *metallica (1)*, *Anopheles sp*. *(1)*, *Not determined (1)*
Unassigned	MP583-NA-2020	*Anopheles sp*. *(1)*, *Cx*. *univittatus (1)*
Unassigned	MP815-NA-2019	*Ma*. *africana (14)*, *Ma*. *uniformis (5)*, *Cq*. *fuscopennata (1)*
Unassigned	MP63-NA-2018	*Aedes sp*. *(3)*, *Cx*. *univittatus (1)*, *Not determined (2)*
Unassigned	MP565-NA-2018	*Not determined (3)*

**Fig 3:**
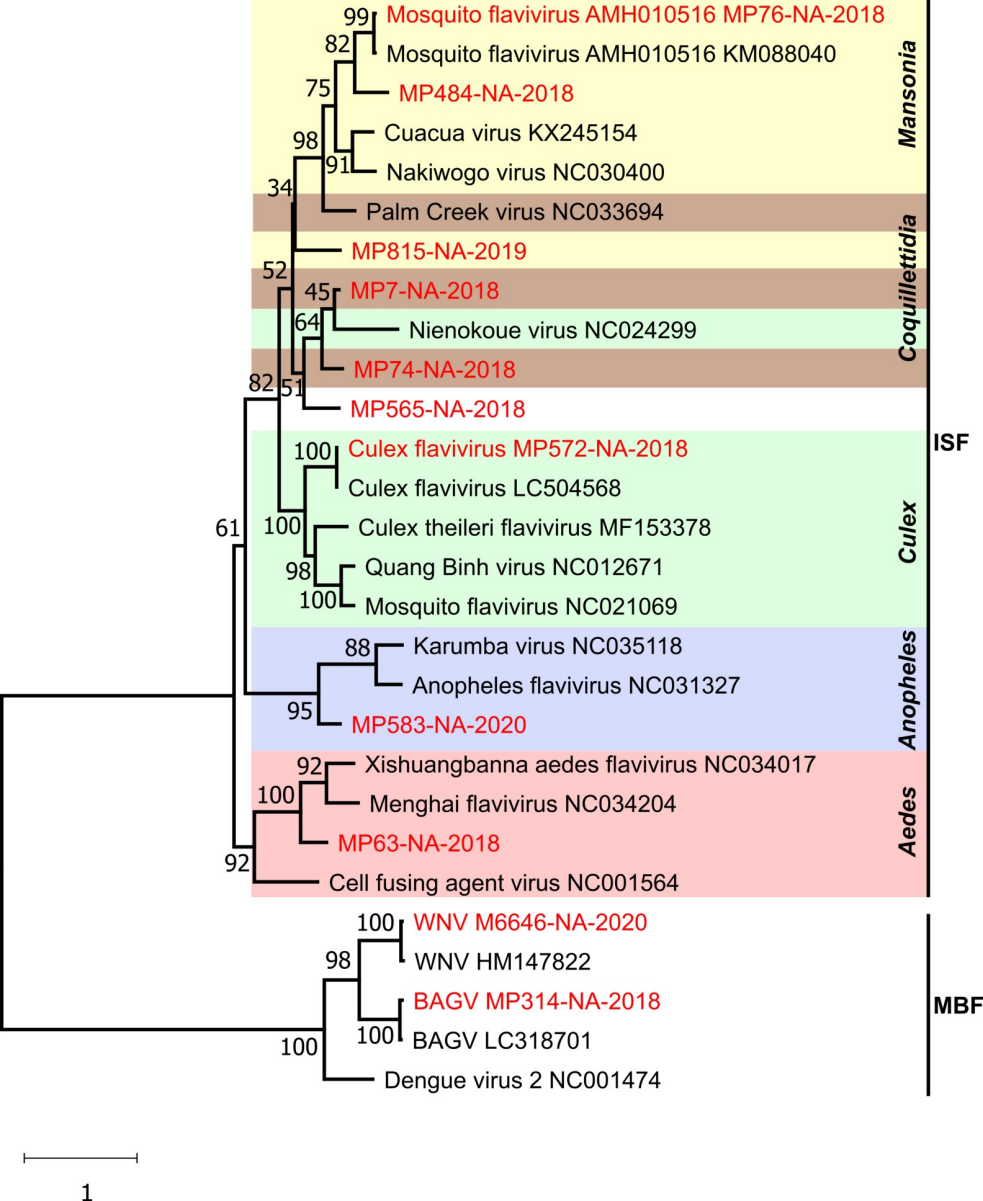
Phylogenetic relationship of flaviviruses detected in the Zambezi region. The phylogenetic tree is based on RdRP gene sequences of 153 nucleotides. Branch support is shown at each branch. Virus sequences detected in this work are shown in red. Gen Bank accession numbers are marked in grey.

Phylogenetic analyses based on one representative strain of every identified virus species and selected flaviviruses confirmed the identification of WNV, BAGV and nine ISFs (Fig 3). WNV M6646-NA-2020 and BAGV MP314-NA-2018 grouped together with Dengue virus 2 (NC001474) in a monophyletic clade comprising mosquito-borne flaviviruses in sister relationship to the clade of ISFs. The Namibian ISFs did not group according to their geographic origin, but rather formed host species associated clades.

### Characterization of WNV

Following the identification of WNV in two mosquito pools, all pools were screened for WNV infections by specific RT-qPCR and individual mosquitoes of the eight WNV-positive pools were tested by specific RT-qPCR. WNV was identified in ten individual female *Cx*. *univittatus* mosquitoes with viral genome copy numbers of 1.3 x 10^5^–6.8 x 10^7^ per mosquito (Table 2). All attempts to isolate the WNV strains from the pooled or individual mosquito homogenates in cell culture failed despite the detection of high viral loads in the mosquitoes. Mosquito species were confirmed by genetic barcoding. We further examined the WNV-positive mosquito extracts (n = 10) for remnants of vertebrate blood meal sources. We identified human mitochondrial DNA in the mosquito that was infected with WNV strain M6848-NA-2020. No vertebrate DNA was found in the other mosquitoes.

**Table 2:** WNV strains identified in the Mashi conservancy in this study.

*WNV strain*	*Host*	*Locality*	*RNA genomic copies per mosquito*	GenBank accession number
M6646-NA-2020	*Cx*. *univittatus*	*Rangeland*	*7*.*94 10*^*6*^	MW383507
M6831-NA-2020	*Cx*. *univittatus*	*Agricultural field*	*6*.*78 10*^*7*^	MW383509
M6848-NA-2020	*Cx*. *univittatus*	*Agricultural field*	*1*.*20 10*^*9*^	MW383508
M7167-NA-2020	*Cx*. *univittatus*	*Agricultural field*	*1*.*35 10*^*5*^	MW436414
M7170-NA-2020	*Cx*. *univittatus*	*Agricultural field*	*1*.*01 10*^*7*^	MW436415
M7238-NA-2020	*Cx*. *univittatus*	*Agricultural field*	*6*.*50 10*^*6*^	MW436416
M7302-NA-2020	*Cx*. *univittatus*	*Agricultural field*	*5*.*86 10*^*7*^	MW436417
M7367-NA-2020	*Cx*. *univittatus*	*Agricultural field*	*3*.*28 10*^*6*^	MW436418
M7373-NA-2020	*Cx*. *univittatus*	*Agricultural field*	*8*.*9 10*^*6*^	MW436419
M7374-NA-2020	*Cx*. *univittatus*	*Agricultural field*	*2*.*48 10*^*7*^	MW436420

**Fig 4:**
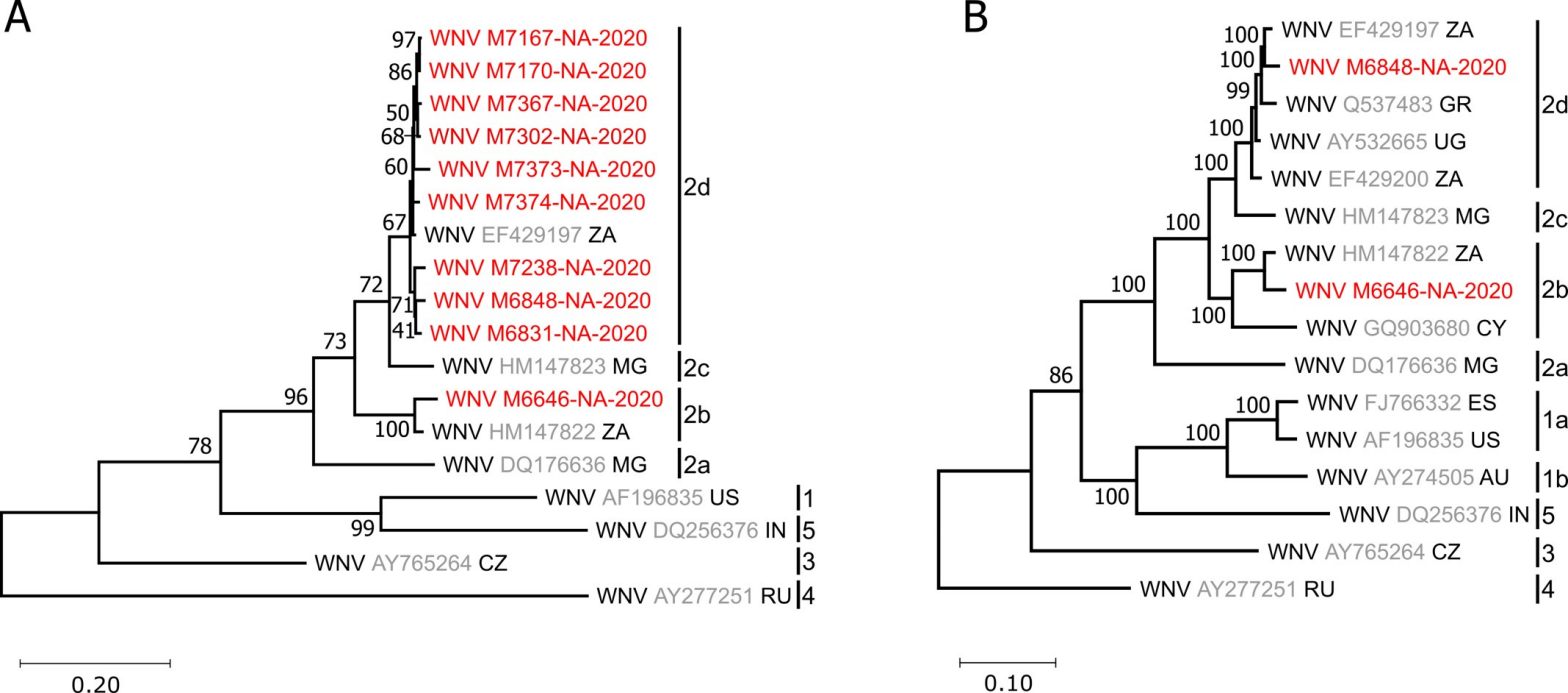
Phylogenetic relationship of detected WNV strains. The phylogenetic tree is based on a 1,032 nucleotide alignment of E-protein sequences of all detected WNV strains and selected global WNV strains (A) or on an alignment of the entire open reading frame (ORF) of WNV M6848-NA-2020 and M6646-NA-2020 and selected global WNV strains (B). Branch support is given for each branch. Virus sequences identified in this study are shown in red. Reference sequences are marked with their accession number in grey and the two-letter code of their countries of origin. AU, Australia; CY, Cyprus; CZ, Czechia; ES, Spain; GR, Greece; IN, India; MG, Madagascar; RU, Russia; UG, Uganda; US, United States of America; ZA, South Africa. The phylogenies are rooted to a WNV lineage 4 strain from Russia (AY277251).

**Fig 5:**
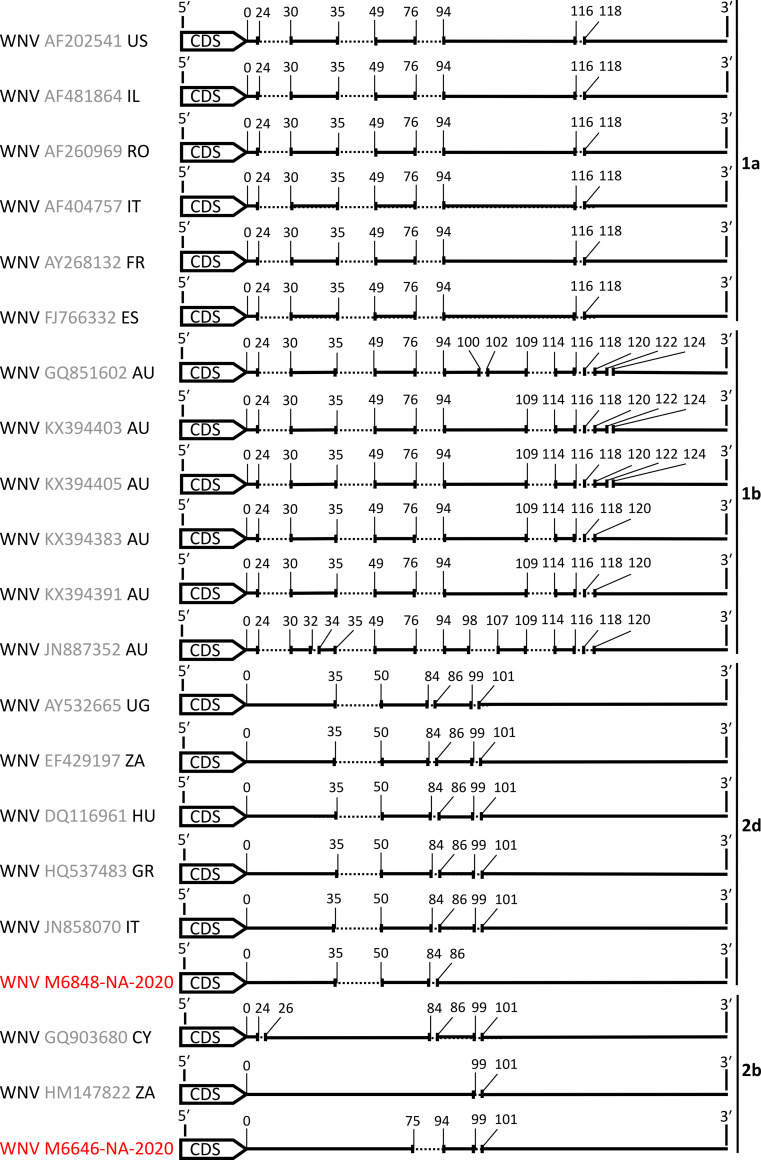
Schematic depiction of WNV 3’ UTR sequences. Strains of different lineages were aligned, and insertions or deletions are shown. Numbers indicate genomic positions. Sequences identified in this study are shown in red.

E-gene sequence analysis and phylogenetic inference revealed a grouping of the ten identified WNV strains with lineage 2 sequences which widely occur across the African continent [67] (Fig 4A). Surprisingly, the detected WNV strains did not group together but fell within two different clades of WNV lineage 2. Nine of the ten WNV strains that originated from the agricultural field clustered with clade 2d, whereas strain M6646-NA-2020 that originated from rangeland grouped with clade 2b. Clade 2b has so far only been detected in South Africa in 1958 and in Cyprus in 1968 [67]. A complete genome of one strain from each clade was sequenced. Phylogenetic analyses based on complete coding sequences confirmed grouping with clades 2b and 2d and showed that M6848-NA-2020 and M6646-NA-2020 each were most closely related to viruses detected in South Africa (Fig 4B). Strain M6646-NA-2020 contained a deletion of 18 nucleotides in the 3’UTR region which was not present in any of the closely related WNV strains (Fig 5).

### Characterization of BAGV

**Fig 6:**
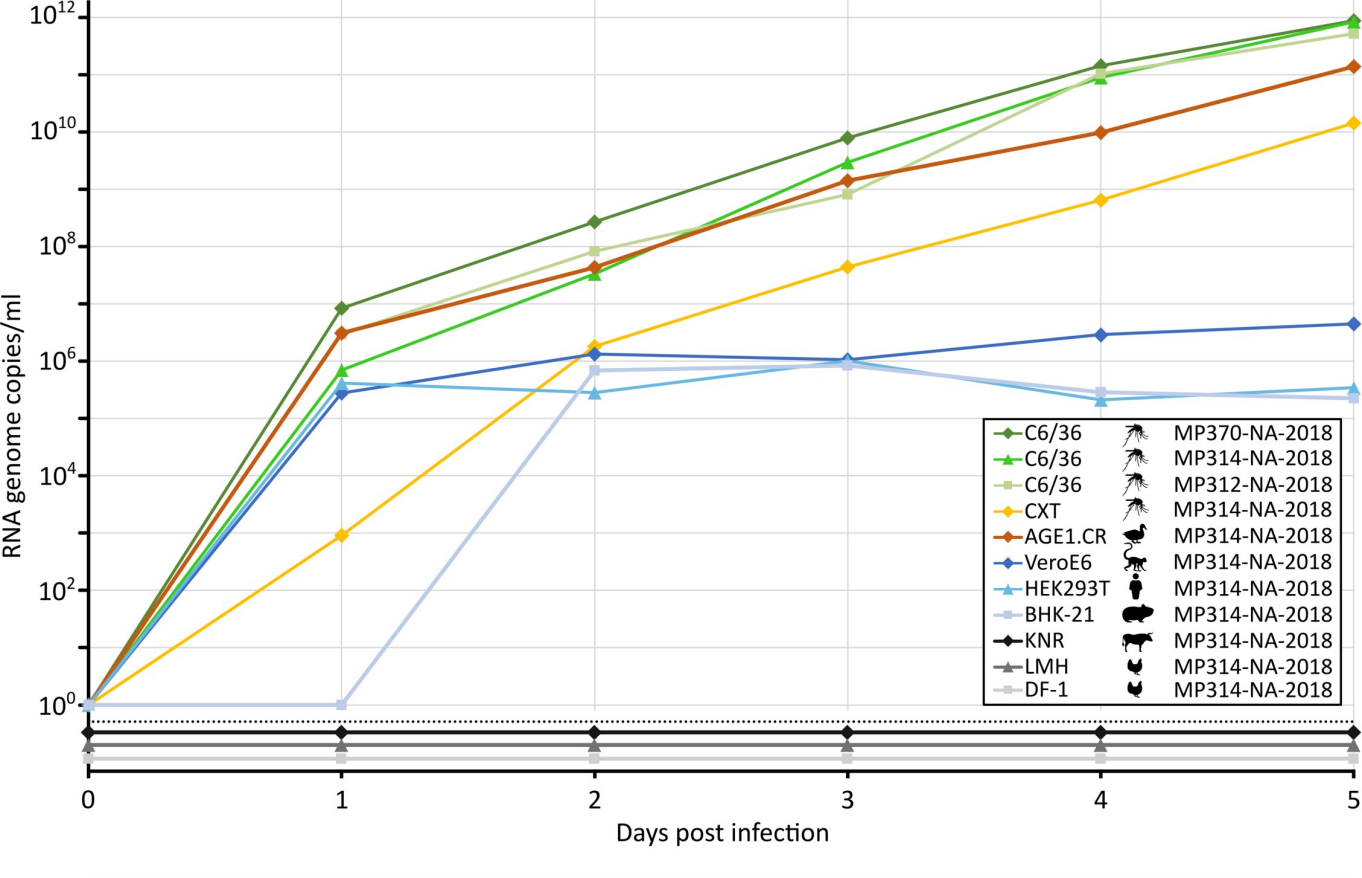
BAGV growth in vertebrate and insect cell lines. The mosquito cell line C6/36 (derived from *Aedes Stegomyia albopictus*) was infected with the three BAGV strains MP312-NA-2018, MP314-NA-2018, and MP370-NA-2018 in duplicates at an MOI of 0.01. Mammalian cell lines BHK-21 (hamster), HEK293T (human), VeroE6 (monkey) and KNR (cattle) were infected in duplicates with BAGV strain MP314-NA-2018 at an MOI of 0.1. Avian cell lines AGE1.CR (duck), LMH (chicken) and DF1 (chicken) and the insect cell line CXT (derived from *Culex tarsalis*) were infected in duplicates with BAGV strain MP314-NA-2018 at an MOI of 0.01. A sample of cell culture supernatant was taken every 24 hours for five consecutive days and viral copy numbers were determined by RT-qPCR.

DNA barcoding showed that the three BAGV-positive mosquito pools consisted of *Cx*. *univittatus* mosquitoes. Virus isolation attempts were successful for all three strains MP312-NA-2018, MP314*-*NA-2018, and MP370-NA-2018 in mosquito cells (C6/36). A CPE was observed in C6/36 cells, 24-, 10-, and 14-days post infection (dpi) for strains MP312-NA-2018, MP314-NA-2018, and MP370-NA-2018, respectively. Growth analyses in C6/36 cells revealed similar growth of the three strains and extremely high genome copy numbers of ca. 1x10^12^ RNA copies/ml five dpi (Fig 6). For further assessment of virus growth and putative host tropism, an array of different cell lines was infected with MP314-NA-2018. The mosquito cell line CXT was also permissive albeit genome copy numbers were approximately two log factors lower compared to C6/36 cells. Since BAGV is a bird pathogen, we infected three different avian cell lines. BAGV was only observed to replicate in AGE1.CR cells derived from muscovy ducks but not in LMH and DF-1 cells derived from chicken. Replication of BAGV in AGE1.CR was almost as productive as in C6/36 cells. BAGV growth was the least-well in mammalian cells with maximum genome copy numbers ca. 10^6^-fold less than in C6/36 cells. However, similar viral RNA copy numbers were reached in non-human primate (Vero), human (HEK293T) and rodent (BHK-21) cells. No growth was observed in cattle-derived cells (KNR).

**Fig 7:**
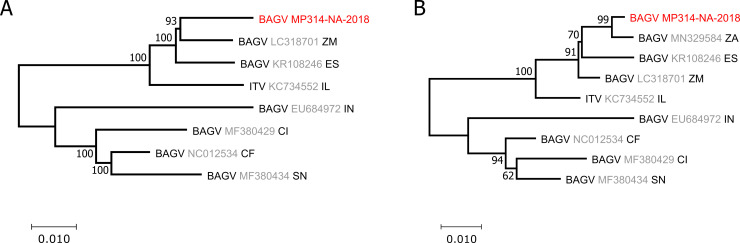
Phylogenetic inference of detected BAGV strains. Phylogenetic relationship of MP314-NA-2018, Israel turkey meningoencephalomyelitis virus (ITV) and selected global BAGV strains based on the entire RdRp gene (A). The tree is based on an alignment of 1,078 nt to include the partial NS5 sequence of a BAGV strain isolated from monal pheasants in South Africa in 2019 (B). Branch support is marked at each branch. Virus sequences detected in this work are shown in red. Reference sequences from GenBank contain the virus name, the accession number in grey and the two-letter code of their countries of origin. CF, Central African Republic; CI, Côte d’Ivoire; ES, Spain; IN, India; IL, Israel; SN, Senegal; ZA, South Africa; ZM, Zambia.

The genome of BAGV strain MP314-NA-2018 was sequenced from infectious cell culture supernatant. The full sequence was uploaded to GenBank under accession number MW672101. Full genome based phylogenetic tree inference showed that BAGV MP314-NA-2018 was most closely related to BAGV strain LC318701 from Zambia (Fig 7A). In further phylogenetic analyses, which included the partial NS5 sequence of a BAGV strain (MN329584) that was isolated from monal pheasants in South Africa in 2016, BAGV MP314-NA-2018 grouped with this sequence (Fig 7B). As MP314-NA-2018 is clearly distinct from LC318701 from Zambia (97.5% divergence) and from MN329584 from South Africa (99.2% divergence based on 1041 nucleotides of viral RdRP sequence), it seems to represent a strain endemic to Namibia.

## Discussion

Information on arbovirus circulation in vector populations in the tropics remain sparse, although surveillance in these regions is of particular importance since arboviruses are primarily endemic in tropical and subtropical regions, often causing local outbreaks there. Here, we screened mosquitoes from the Zambezi region of north-eastern Namibia for infection with flaviviruses and report the detection of the vertebrate-pathogenic arboviruses WNV and BAGV, as well as the identification of 241 strains of two previously known and seven putative novel insect-specific flaviviruses. This is the first detection of WNV and BAGV in insect vectors in Namibia and also the first detection of WNV in Namibia in more than 30 years.

We detected WNV at the end of the yearly rainy season in March 2020 but not at the end of 2018 nor during or at the end of the rainy season in 2019. Heavy precipitation is a key driver for an upsurge in mosquito populations and WNV infections, especially in dryer landscapes where mosquitoes only find breeding places after heavy rainfall and in the wet season [68]. Such heavier rainfalls may have contributed to the detection of WNV in 2020. With 253.9 mm precipitation in the Zambezi region was notably higher between January and March 2020 compared to the 98.3 mm and 76.1 mm recorded for the same time period in 2018 and 2019, respectively [69].

WNV is transmitted by a large variety of mosquito species [70]. Mosquitoes of the *Culex pipiens* L complex are the primary WNV vectors in North America and Europe. In contrast, *Cx*. *univittatus* has been identified as the most important WNV vector in South Africa [71]. We exclusively detected WNV in female *Cx*. *univittatus* mosquitoes, confirming the importance of this species as WNV vector in southern Africa. Of note, WNV was only found in the Mashi conservancy in 2020 but not a single WNV infection was detected in Mudumu National Park and Wuparo Conservancy in 2018 where the absolute numbers of collected *Cx*. *univittatus* mosquitoes peaked (Fig 2). Such a discrete temporal and spatial distribution pattern of WNV has been observed in long term surveys in the USA [72].

We observed a WNV prevalence of 0.1% (10/10,206 mosquitoes). Other studies conducted in South Africa between 1956 to 1980 found WNV infection rates in mosquitoes of less than 0.026% [71]. However, infection rates in *Cx*. *univittatus* can reach up to 39.0/1000 or 9.6/1000 during WNV epizootics in the same region [71]. Notably, all the ten detected WNV strains fell into two different lineage 2 clades, with one strain grouping with clade 2b and nine strains grouping with 2d. The 2b strain (M6646-NA-2020) originated from rangeland and the 2d strains from agricultural fields in the Mashi conservancy that were about 9 km apart from each other, further emphasizing that WNV is endemic and diversified in Namibia. Identity values of 95.9–99.9% in pairwise alignment of partial E-protein fragments of the nine 2d sequences support a genetically diversified set of strains. This becomes particularly evident when comparing the observed genetic divergence rate of maximal 4% to that found in other studies. For example, nucleotide divergences in mosquitoes were found to range between 1–2% in the Montréal area of Canada from 2004–2016 [73], and below 1% in the US, 0.41%–0.72% in Texas [74], 0.26%–0.5% in Chicago [75], and 0.3% in Connecticut [76]. Higher divergence rates of 0.16–3% were found in mosquitoes from Israel [77]. Overall, the detection of WNV strains from two clades and the observed genetic nucleotide divergence of about 4% in 10 mosquitoes from one area at a single time point demonstrate the circulation of a genetically diverse WNV population in the Zambezi region of Namibia.

We could further show that the mosquito specimen that was infected with M6848-NA-2020 took a blood-meal on a human host indicating human exposure to WNV. We could not identify any vertebrate DNA remaining from blood feeding in the other nine WNV-positive mosquitoes. Although *Cx*. *univittatus* is primarily ornithophilic, it has been shown to also feed on humans and was found in human dwellings [71]. Maintenance of WNV in its enzootic cycle in southern Africa involves ornithophilic mosquitoes and a wide range of southern African avian species [71]. Taken together, the high WNV detection rate and genetic diversity, as well as human host sources indicate the public health relevance of WNV in Namibia. However, there is a general lack of knowledge on the ecology and epidemiology of WNV in Africa including the study region of this paper. Other knowledge gaps include unknown distribution and incidence patterns of WNV in Africa, as well as on species involved in its natural amplification cycles. Future studies should focus on endemic transmission cycles and geographic distribution of WNV, as well as human infection rates with WNV.

To our knowledge, so far only two other clade 2b strains have been detected. One was found in a bird in South Africa in 1958 and the other in a bird in Cyprus in 1968 [67]. We discovered an 18-nucleotide deletion in the 3’ UTR of strain M6646-NA-2020 that was not present in the other strains of clade 2b. The 3’UTR region of WNV is known to harbor a variety of sequence elements that play a role in maintenance of correct viral RNA secondary structures, interaction with cellular proteins and protecting the viral genome from degradation by RNases [78,79]. These highly conserved, functional regions are located in the distal part of flavivirus UTRs, close to the genome 3’-end. However, the downstream part of the flaviviral 3’ UTR that closely follows the 3’-end of the CDS, is well known for its high variability and typically contains insertions and deletions [80].

Unfortunately, we were not successful in isolating any of the WNV strains in cell culture although genome copies of up to 6.8 x 10^7^ per mosquito were found. This is most probably due to thawing of the mosquito samples during their transport to the laboratory in Germany as the dryshipper was damaged on the flight. In contrast, all detected BAGV strains were successfully isolated in cell culture from mosquitoes. These samples were transported previously, and no damage was observed.

Three BAGV strains were uncovered in *Cx*. *univittatus* mosquitoes from Mudumu National Park in the Zambezi region. BAGV has previously been isolated from several species of the *Culex* genus [32,34,35] including *Cx*. *univittatus* [33]. BAGV is maintained in nature by transmission between wild birds of the Phasianidae family and *Culex* spp. [81]. Susceptible species also include farmed game birds like turkeys and wild game birds like pheasants and partridges [26–28,31]. Several members of the Phasianidae family, like *Peliperdix coqui*, *Scleroptila gutturalis*, *Dendroperdix sephaena*, *Pternistis adspersus*, and *Pternistis swainsonii* are endemic in north-eastern Namibia [82]. However, little is known on BAGV maintenance in the Zambezi region. Screening of potential natural bird reservoir species for antibodies against BAGV could provide further insight into endemic transmission cycles, geographic distribution, and virus prevalence. In this study, growth analyses revealed great differences between cells derived from hosts involved in the natural virus transmission cycle, mosquito and bird cell lines, and mammalian cell lines derived from primates, rodents, cattle, and humans. Viral genome copy numbers were on average 100,000 times higher in mosquito and bird cells than in mammalian cells, except for cattle cells which were not susceptible. The mammalian cell lines BHK-21 and VeroE6 have been reported to support BAGV replication [30]. We found that the human cell line HEK293T enabled BAGV replication to a similar extent than BHK-21 and VeroE6 cells. Possibly BAGV is capable of infecting humans as neutralizing antibodies were found in encephalitic patients from India [36]. Such susceptibility of human cells for BAGV infection would indicate that humans may not be refractory for BAGV infection. We further found that muscovy duck cells *(Cairina moschata*, Anatidae) support BAGV replication to similar levels as mosquito cells, whereas chicken-derived cells were not susceptible. Although muscovy ducks are endemic to Central America, these findings may imply that BAGV may infect a wider range of bird species than partridges and pheasants. In Spain common wood pigeons (*Columba palumbus*, Columbidae) can also be infected by BAGV [83]. Thus, testing a wide variety of different bird species of the Zambezi region will help to identify the susceptible species and provide insight into virus maintenance in nature.

The high detection rate of insect-specific flaviviruses (95%, 241 of 254 pools positive) in the sample set confirmed findings from other studies, providing further evidence that ISFs are widely spread in mosquito populations worldwide, now including southern Africa [84–87]. Co-infection studies have shown that infection with insect-specific viruses can alter replication and transmission of arboviruses in mosquitoes [88]. Notably, an inhibition of WNV replication mediated by a variety of ISFs in mosquitoes has been reported [89–92]. In contrast, Culex flavivirus, that was also found in mosquitoes of this study (Fig 3), seems to be positively associated with WNV infections in mosquitoes [93]. We have not observed any co-infections with multiple flaviviruses in our samples. However, *in vitro* co-infection experiments with arboviruses and ISFs detected in this study could identify if these viruses could have an effect on vector competence or transmission rates.

In conclusion, we demonstrated that a wide genetic diversity of flaviviruses is circulating in mosquitoes in the Zambezi region of north-eastern Namibia. Importantly, we identified two clusters of WNV belonging to two different clades and BAGV. WNV and BAGV can cause outbreaks, including severe disease and mortality in humans and birds, respectively. Further studies are needed to assess their health impact, as well as their social and economic impact in southern Africa.

## Supporting information

S1 Table Primers used for BAGV genome amplification. (DOCX)
