# Supplementary material for: Simultaneous circulation of two West Nile virus lineage 2 clades and Bagaza virus in the Zambezi region, Namibia
Source: PLoS Negl Trop Dis. 2021 Apr 2;15(4):e0009311. doi: 10.1371/journal.pntd.0009311 (PMC8046352; doi:10.1371/journal.pntd.0009311)
Supplement: S1 Table — (DOCX) [file pntd.0009311.s001.docx]

**Supplementary Material:**

**Table S1:** Primers used for BAGV genome amplification.

| Primer name | Primer sequence (5’ – 3’) |
| --- | --- |
| BAGV F1 | TGACAGCTCAACACAAGTGC |
| BAGV R1 | CTGGTTGACTGTGACAAGTGG |
| BAGV R1n | CCTGAGCCACCCCTAACAC |
| BAGV F2 | AGTAGGGCCAGTCAGAATCG |
| BAGV F2n | CTTTGAAACCAACGGCAGGA |
| BAGV R2 | ATGAGCCAGCAGATGATCCA |
| BAGV R2n | TGTTCACAAGCCAACTCTTCG |
| BAGV F3 | CCGAAAGCACCAGTGTACAC |
| BAGV F3n | CTCTGGGATGGACATGAGCC |
| BAGV R3 | TGCTTTGTCGCCCTTAATGG |
| BAGV R3n | CCTTAGTCCTAACCAAACTTTCG |
| BAGV F4 | TCACTCCACCCATGTCCAAT |
| BAGV F4n | GCCCAGATGAGAATAGAGCCT |
| BAGV R4 | CCCATCACAATCTATCTCAACGT |
| BAGV R4n | CTCCAGCCACAAGTCAGTCG |
| BAGV F5 | AGTGAGTGAAGCCCTTACGG |
| BAGV F5n | GGGATCGCACACTTTGACG |
| BAGV R5 | TGCATTTCCGACTGTCAATGA |
| BAGV R5n | CAAAGTCCCATTCGCCTGTT |
| BAGV F6 | AAGACAGTGTGGTTTGTTCCT |
| BAGV F6n | GTGTGTCTGACGAAGGCC |
| BAGV R6 | TTGCCACGATTCCATCAACC |
| BAGV R6n | CCAGCCGGGGATCAGAAA |
| BAGV F7 | TTGGTTTGGGAAAGGGCATG |
| BAGV F7n | ACTGTTAGGGTGTTGGGGTC |
| BAGV R7 | TGAACTCCTCCTTTGTGCAGA |
| BAGV R7n | CATCCATCGGGCTGTGATTT |
| BAGV F8 | AAACCTTGGGACGCCTTACA |
| BAGV F8n | GACCGACACAACCCCATTTG |
| BAGV R8 | CGGTTCAGAGTTGGCATGTAG |
| BAGV R8n | GGGCTCTGATCTGCATAATTGG |
| BAGV F9 | GGGTGTGGATTGAGGAGAATG |
| BAGV F9n | CACATGGAAGACAAGACCCC |
| BAGV R9 | GTGTTCTACCACCACCAGC |
